# Comparison of cancer survival in UK and Australia: rates are higher in Australia for three major sites

**DOI:** 10.1038/sj.bjc.6602154

**Published:** 2004-10-12

**Authors:** X Q Yu, D L O'Connell, D Forman

**Affiliations:** 1 Cancer Epidemiology Research Unit, The Cancer Council New South Wales, Sydney, Australia; 2 Northern and Yorkshire Cancer Registry and Information Service, Arthington House, Cookridge Hospital, University of Leeds, Leeds, UK; 3 Unit of Epidemiology & Health Services Research, Medical School, University of Leeds, UK

**Keywords:** relative survival, statistical methods, cancer survival, cancer registries

## Abstract

Relative survival of patients diagnosed with cancers of the colorectum, lung and female breast from Yorkshire, UK and New South Wales (NSW), Australia in 1992–2000 were compared using multiple regression models to adjust for various factors. Statistically significant differences were observed for all sites, Yorkshire patients having a 47–58% higher risk of excess death than those of NSW.

UK cancer survival rates have been reported to be inferior to those from many other European countries for most of the common cancer types (Sant *et al*, 2003). The EUROCARE data, on which such reports have been based, have been criticised from several perspectives (Cookson, 2000; Woodman *et al*, 2001), among which have been concerns that the processes of cancer registration have not been strictly comparable. Cancer registration and mortality notification systems are directly comparable in the UK and Australia, and routine published survival data show substantial differences between the two countries with outcomes generally being worse in the UK (Australian Institute of Health and Welfare (AIHW) and Australasian Association of Cancer Registries, 2001; National Statistics, 2002).

Routine cancer survival rates can, however, be difficult to compare due to variation in the methodological and statistical approaches used. We have compared, therefore, survival rates for three common cancers (colorectal, lung and female breast) in Yorkshire, UK and New South Wales (NSW), Australia using identical methodology in an analysis of individual patient data for cases diagnosed from 1992 to 2000. The cancer registries providing the data (the Northern and Yorkshire Cancer Registry and Information Service – NYCRIS and the NSW Central Cancer Registry – NSWCCR) are population based, have been operational for several decades and have high standards of data completeness, quality and follow-up. The population sizes for the two registries are large: 3.6 million for the Yorkshire data within NYCRIS and 6.5 million for NSWCCR, and rates of cancer survival in the registry populations are similar to the corresponding national rates for the UK and Australia.

## MATERIALS AND METHODS

Data sets with individual patient records were provided by NYCRIS and NSWCCR for all new diagnoses of cancer of the colorectum, lung and female breast during the time period 1992–2000. Follow-up for death was complete in both registries until 31 December 2001. Only first occurrences of primary cancer for individuals aged 15–89 years at diagnosis were included. Cases notified by death certificate only, identified at postmortem or with a survival time equal to zero (diagnosed and died on the same day), were excluded from analyses.

A modification of the period method (Brenner and Gefeller, 1996) was used to compute 5-year relative survival ratios (RSR) of cancers diagnosed in the period 1992–2000 and deaths 1996 to 2001. Survival time was measured from the month of diagnosis to the date of death, or in the period at the end of 2001. The period method focuses on a recent time interval (1996–2001) in ensuring which each patient's survival experience is observed and excludes short-term survival of patients diagnosed before the start of the interval (diagnosed 1992–1995 and dying before 1996) but includes their long-term survival within the period. Short-term survival of more recently diagnosed patients (those diagnosed between 1996 and 2000) was included. Observed and expected survival were estimated using standard life table methods (Voutilainen *et al*, 2000). Life tables for the relevant populations were obtained from the (England and Wales) Office for National Statistics for the Yorkshire and Humber Region (1998) and from the Australian Bureau of Statistics for NSW (1996–2000). A 5-year cumulative RSR was calculated as the ratio of the cumulative observed survival to the cumulative expected survival, with 95% confidence intervals (CI) calculated using the complementary log–log transformation (Voutilainen *et al*, 2000). Age-standardised RSR were calculated using the EUROCARE method (Berrino *et al*, 1995) and the corresponding 95% CI were computed using a logistic transformation.

A Poisson regression model (Dickman *et al*, 2004) of excess risk of death during the first 5 years was constructed for each type of cancer, which included age group (15–44, 45–59, 60–74 and 75–89 years), years since diagnosis, sex (for lung and colorectal cancers) and place of residence (Yorkshire/NSW) as the main effects, and the interaction between age group and years since diagnosis to allow for nonproportional hazards across years of follow-up. The relative excess risk (RER) of death for each cancer type for Yorkshire was defined as the ratio of the excess risk of death in Yorkshire to that in NSW. The RER and its 95% CI were calculated using the estimated coefficients and standard errors from the Poisson model. The effect of place of residence on the excess risk of death was tested using the likelihood ratio statistic with *P*<0.01 indicating statistical significance.

## RESULTS

Table 1

**Table 1:** The 5-year observed and RSR, age-standardised 5-year RSR and RER of death for three major cancers in Yorkshire, UK and NSW, Australia, patients diagnosed in 1992–2000 and with mortality in 1996–2001

	**Number of new cases**		**5-year observed survival (%)**		**5-year RSR (%) with (95% CI)**		**Age-standardised 5-year RSR (%) with (95% CI)**		
**Cancer type**	**Yorkshire**	**NSW**	**Yorkshire**	**NSW**	**Yorkshire**	**NSW**	**Yorkshire**	**NSW**	**RER^a^ for Yorkshire (95% CI)**
Colorectal	16 710	27 801	38.3	52.5	49.3 (48.2–50.4)	60.5 (59.7–61.3)	50.3 (48.4–52.2)	60.1 (58.9–61.3)	1.47 (1.42–1.53)
Lung	15 864	14 816	5.6	12.1	6.9 (6.4–7.3)	13.8 (13.2–14.4)	7.7 (5.9–9.9)	13.1 (11.4–15.0)	1.49 (1.45–1.53)
Breast (F)	20 338	28 684	68.9	79.6	77.2 (76.3–78.1)	84.9 (84.3–85.5)	78.2 (77.0–79.3)	84.5 (83.8–85.3)	1.58 (1.48–1.68)

RSR=relative survival ratios; RER=relative excess risk; 95% CI=95% confidence interval.

a Adjusted for age at diagnosis, sex, years since diagnosis and interaction of age and years since diagnosis in a Poisson regression model.

shows, for each site of cancer, the number of cases included in the analysis, observed and relative 5-year survival and age-standardised 5-year RSR in the two populations, together with RER of death due to cancer for Yorkshire relative to NSW. Statistically significant differences in 5-year RSR were found for all three types of cancer with NSW patients consistently having better outcomes. There were benefits in RSR after age standardisation of more than 5% for lung and female breast cancers and of almost 10% for colorectal cancer. Statistically significant differences (*P*<0.0001) in survival for all three cancer types were observed after adjustment for age, sex and years since diagnosis with the RER of death being 47–58% higher in Yorkshire patients.

## DISCUSSION

This study has shown moderate but important differences in cancer survival between the UK and Australia. For all three cancers, colorectal, lung and female breast, the 5-year survival rates were lower in the UK population with a statistically significant RER of death of around 50%. The rates were based on substantial numbers of cases and deaths and represent cancers with relatively good (breast), moderate (colorectal) and poor (lung) prognoses. We have used identical statistical methodology to analyse the two data sets and age-adjusted comparisons to control for the younger age structure of the Australian population.

Both NYCRIS and NSWCCR fulfil the requirements of a mature cancer registry (Woodman *et al*, 2001): total population coverage, with notifications from multiple sources to maximise case ascertainment, and efficient and regular linkage to death certificates. We believe, therefore, that the data quality and processes of registration of the two registries are similar and comparable and that the observed differences in survival cannot readily be attributed to variations in the validity of the registry data. In neither population was there any systematic screening for colorectal or lung cancers during 1992–2000 and both populations had similar levels of breast cancer screening. In Yorkshire, 75.2% of women aged 50–64 years accept first invitations to screening (86.9% after initial screen), with 4.9 cancers detected per 1000 women screened (NHSBSP, 2003), whereas in NSW initial screening rates are 66.8% (80.1% for subsequent screen) for women aged 50–69 years, with 5.4 cancers detected per 1000 women (Estoesta *et al*, 2000). Intensity of screening activity is, therefore, unlikely to be an explanation for the differences.

The most recently published EUROCARE results (Sant *et al*, 2003) cover an earlier time period (1990–1994) than that considered here, but it is worth noting that the survival differences between Yorkshire and NSW that we have reported are broadly similar to those between England and the European weighted mean reported in EUROCARE. As with EUROCARE, the differences in survival between the populations may represent differences in access to, and quality of care delivered, earlier diagnosis, or a combination of these variables. These factors can only be disentangled by more detailed studies that consider both the stage of disease at presentation and the treatment received after diagnosis.
